# MOG antibody-associated optic neuritis

**DOI:** 10.1038/s41433-024-03108-y

**Published:** 2024-05-23

**Authors:** Niroshan Jeyakumar, Magdalena Lerch, Russell C. Dale, Sudarshini Ramanathan

**Affiliations:** 1 Translational Neuroimmunology Group, Kids Neuroscience Centre and Sydney Medical School, Faculty of Medicine and Health, University of Sydney, Sydney, NSW Australia; 2 Department of Neurology, Westmead Hospital, Sydney, NSW Australia; 3 Brain and Mind Centre, Faculty of Medicine and Health, University of Sydney, Sydney, NSW Australia; 4 Clinical Neuroimmunology Group, Kids Neuroscience Centre and Sydney Medical School, Faculty of Medicine and Health, University of Sydney, Sydney, NSW Australia; 5 TY Nelson Department of Neurology, Children’s Hospital at Westmead, Sydney, NSW Australia; 6 Department of Neurology, Concord Hospital, Sydney, NSW Australia

**Keywords:** Optic nerve diseases, Eye manifestations

## Abstract

Myelin oligodendrocyte glycoprotein (MOG) antibody-associated disease (MOGAD) is a demyelinating disorder, distinct from multiple sclerosis (MS) and neuromyelitis optica spectrum disorder (NMOSD). MOGAD most frequently presents with optic neuritis (MOG-ON), often with characteristic clinical and radiological features. Bilateral involvement, disc swelling clinically and radiologically, and longitudinally extensive optic nerve hyperintensity with associated optic perineuritis on MRI are key characteristics that can help distinguish MOG-ON from optic neuritis due to other aetiologies. The detection of serum MOG immunoglobulin G utilising a live cell-based assay in a patient with a compatible clinical phenotype is highly specific for the diagnosis of MOGAD. This review will highlight the key clinical and radiological features which expedite diagnosis, as well as ancillary investigations such as visual fields, visual evoked potentials and cerebrospinal fluid analysis, which may be less discriminatory. Optical coherence tomography can identify optic nerve swelling acutely, and atrophy chronically, and may transpire to have utility as a diagnostic and prognostic biomarker. MOG-ON appears to be largely responsive to corticosteroids, which are often the mainstay of acute management. However, relapses are common in patients in whom follow-up is prolonged, often in the context of early or rapid corticosteroid tapering. Establishing optimal acute therapy, the role of maintenance steroid-sparing immunotherapy for long-term relapse prevention, and identifying predictors of relapsing disease remain key research priorities in MOG-ON.

## Abstract (zh)

髓鞘少突胶质细胞糖蛋白 (MOG)抗体相关疾病(MOGAD)是一种脱髓鞘疾病, 有别于多发性硬化症 (MS) 和视神经脊髓炎视神经疾病 (NMOSD) 。MOGAD最常表现为视神经炎 (MOG-ON), 通常具有特征性的临床和影像学特征。MOGAD双侧受累、临床和影像学表现为视盘肿胀以及纵向MRI上广泛视神经高信号伴相关视神经束膜炎是有助于区分MOG-ON与其他病因所致视神经炎的关键特征。在具有相同临床表型的患者中, 利用基于活细胞的测定法检测血清MOG免疫球蛋白G对于MOGAD的诊断具有高度特异性。本综述将重点介绍加快诊断的关键临床和影像学特征及辅助检查, 如视野, 视觉诱发电位和脑脊液分析。这些辅助检查的诊断价值相对较弱, OCT可以识别急性视神经肿胀和慢性视神经萎缩, 并可能作为诊断和预后的生物标志物。MOG-ON似乎对皮质类固醇较敏感, 皮质类固醇通常是急性期治疗的一线药物。然而, 在随访时间延长的患者中很容易复发, 通常是在早期或快速减少皮质类固醇的情况下。最佳策略的的急性期治疗, 维持类固醇保留免疫疗法在长期预防复发中的作用, 以及确定疾病复发的预测因子仍然是MOG-ON的关键研究重点。

## Introduction

Myelin oligodendrocyte glycoprotein antibody-associated disease (MOGAD) is a demyelinating disorder of the central nervous system (CNS) distinct from multiple sclerosis (MS) and aquaporin 4 (AQP4) immunoglobulin G (IgG) antibody-associated neuromyelitis optica spectrum disorder (NMOSD). MOGAD refers to a demyelinating syndrome in association with IgG autoantibodies targeting myelin oligodendrocyte glycoprotein (MOG), a minor transmembrane surface protein found on the outermost lamellae of CNS myelin and oligodendrocytes [1, 2]. The annual incidence of MOGAD worldwide is approximately 1.6–4.8 per million people, with a prevalence estimated at 1.3–2.5 per 100,000 people [3–5]. There is a biphasic distribution of the age of onset, peaking in children aged 5–10 years as well as in adults aged 20–45 years. The median age of onset overall is 20–30 years [6, 7]. MOGAD accounts for nearly 50% of acute demyelinating syndromes in children under 11 years of age [8]. Optic neuritis (ON) is the most common initial manifestation of MOGAD in adults (~30–60%), followed by transverse myelitis (~10–25%), and is the second most common manifestation in children, after acute disseminated encephalomyelitis (ADEM; ~45% in children <11 years of age vs. <5% in adults) [1, 9]. This review aims to summarise current knowledge on MOG antibody-associated optic neuritis (MOG-ON), from presentation to diagnosis and treatment, in order to assist clinicians in managing this increasingly recognised condition.

## Historical evolution and pathophysiology of MOGAD in human demyelination

Early studies of MOG IgG were hampered by the use of Western blots and enzyme linked immunosorbent assays (ELISAs), which denature and linearise the MOG peptide respectively, while it was identified that only MOG IgG that binds to MOG in its native conformational state is potentially pathogenic [10–16]. O’Connor et al., using a MOG tetramer radioimmunoassay, first identified the presence of MOG-IgG in children presenting with ADEM [17]. Since then, it has been the advent of live cell-based assays, which has enabled native human MOG to be transduced or transfected into a mammalian cell line for surface expression, incubated with sera and a secondary anti-human IgG antibody, and analysed qualitatively (with microscopy) or quantitatively (with flow cytometry) for MOG-specific antibody binding [18–22]. This methodology has enabled the detection of clinically relevant MOG-IgG and accurate identification of children and adults with a demyelinating syndrome distinct from MS and NMOSD.

Histopathological data on MOGAD in humans is limited. MOGAD is an oligodendrogliopathy rather than an astrocytopathy like NMOSD, where the antigen is AQP4 expressed on astrocytic end feet. The immune response in MOGAD-related inflammatory plaques appears to be dominated by CD4-positive T cells with lesser infiltration of CD8-positive T cells and B cells [23]. Other histopathological features include variable infiltration of granulocytes, MOG-laden macrophages, some complement and Ig deposition in active white matter lesions, variable oligodendrocyte and axonal destruction, astrogliosis, and some overlapping features with MS and ADEM [23, 24]. There are divergent findings regarding whether or not there is preferential loss of MOG [23, 24]. Moreover, pre-myelinating oligodendrocytes are sometimes visible without signs of active remyelination [23].

The CSF profile of MOGAD patients can display elevated levels of both B cell and T cell-related pro-inflammatory cytokines and chemokines, supporting complex innate and adaptive immune activation [25]. Some of these cytokines, such as IL-6, have potential as therapeutic targets. Indeed, the off-label use of tocilizumab (an IL-6 receptor antibody) in MOGAD has been shown to prevent relapses over a duration of up to 29 months [26].

One study observed specific B-cells in the periphery of MOGAD patients, though a correlation with MOG-IgG titres was absent [27]. More detailed analyses revealed increased memory B-cells and T-follicular helper cells, as well as decreased regulatory B-cells, in MOGAD [28]. The search for MOG specific T cells in MOGAD has been challenging, and stimulation of peripheral blood mononuclear cells with different MOG peptides has not been shown to lead to antigen specific responses thus far [29].

Antibodies taken from the sera of patients with MOG-ON, which are primarily of the IgG1 isotype, induce demyelination in animal models with involvement of the complement pathway if they are cross-reactive with rodent MOG in combination with myelin basic protein-specific T cells; together with MOG-specific T cells, they evoke enhanced T cell infiltration [30]. Studies examining the potential for MOG-IgG to activate the complement system in humans made divergent observations and different complement-independent mechanisms have also been demonstrated [31, 32].

The trigger for antibody production in MOGAD is unknown. As with many other cell surface antibody-associated CNS disorders, it is believed that a break in immune tolerance may be triggered in the peripheral circulatory compartment, with subsequent passage of immune cells into the intrathecal compartment and the CNS, and ongoing trafficking of immune cells between the brain parenchyma, CSF and circulation [33]. Cases of vaccination and infection preceding MOGAD are documented [34–36]. These may trigger the autoimmune cascade by mechanisms such as bystander activation, whereby chemokines and cytokines released in the immune response to a foreign pathogen activate autoreactive lymphocytes; or, less likely as the infectious agent reported in the literature varies, molecular mimicry, whereby similarities between foreign and self-antigens activates autoreactive lymphocytes. Other systemic autoimmune disorders appear to be uncommon in MOGAD, unlike in NMOSD [37]. Cancer diagnoses associated with MOGAD presentations are very rare (<1%), and likely represent the background malignancy risk rather than a paraneoplastic association [37]. Tumour necrosis factor alpha inhibitor therapy has been rarely associated with the development of MOGAD [38].

The pathophysiology of ON in MOGAD is particularly interesting as MOG is not expressed in the retina[39]. Other mechanisms have been proposed to contribute to retinal ganglion cell degeneration, such as glutamate cytotoxicity [40], as well as the lack of classical blood-brain barrier characteristics of the optic nerve head [41].

## Clinical presentation of MOG-ON

MOG-ON presents with typical symptoms of acute ON such as pain and loss of visual acuity. However, certain features, such as bilateral involvement, optic disc swelling and optic perineuritis are characteristic and favour the diagnosis of MOG-ON over other demyelinating aetiologies such as NMOSD and MS. Table 1 highlights the commonalities and differences between ON associated with these three key aetiologies with respect to demographics, clinical and radiological characterisation, investigations, and therapeutic and prognostic features.

**Table 1:** Comparison of MOG-ON, AQP4-ON and MS-ON.

Characteristic	MOG-ON	AQP4-ON	MS-ON
**Demographics**			
Age at onset	Paediatric and adult onset	Adult onset Paediatric onset rare	Adult onset Paediatric onset less common
Sex assigned at birth (F:M)	1:1	7–9:1	3:1
Ethnic propensity	Caucasian	Afro-Caribbean	Caucasian
Disease course	Monophasic or relapsing	Most often relapsing	Relapsing Secondary progressive Infrequently primary progressive
**Clinical assessment**			
Clinically bilateral at onset	Frequent	May be present	Extremely rare
Associated headache	Frequent	Rare	Rare
Visual acuity at nadir	Moderate - severely impaired	Moderate - severely impaired	Mild - moderately impaired
Fundoscopy at nadir	Moderate - severe optic disc swelling is common; may have associated haemorrhages	May be present	Mild optic disc swelling may be present; rarely moderate-severe
Initial recovery	Typically favourable, particularly with corticosteroids	May be poor	Typically favourable
**Radiological characterisation**			
Radiological optic nerve head swelling	May be present	Rare	Rare
Longitudinally extensive optic nerve involvement (>½ length of optic nerve)	Frequent	Frequent	Rare; typically focal optic nerve involvement
Optic nerve sheath involvement (optic perineuritis)	Frequent	Rare	Not reported
Optic nerve oedema	Frequent	Frequent	May be present
Optic chiasmal involvement	Less frequent (unless extension of contiguous longitudinally extensive optic neuritis)	May be present	Rare
Optic tract involvement	Rare	May be present	Rare
Visual pathway involvement	Frequently anterior	Frequently posterior	Frequently anterior
**Ancillary investigations**			
Visual fields	Variable Peripheral vision loss in optic perineuritis	Variable	Variable
OCT			
Acute	pRNFL thickening; may be significantly swollen	pRNFL thickening	pRNFL thickening
Follow-up	pRNFL thinning (MOG > MS)	pRNFL thinning	pRNFL thinning
CSF			
Pleocytosis	Frequent; can be very high (>50 cells/μL), especially if accompanying myelitis	Frequent; can be very high (>50 cells/μL)	Variable; rarely very high
Elevated protein	May be present	May be present	Less common
Oligoclonal bands	May be present	May be present	Very frequent
**Therapeutic and prognostic features**			
Rapid steroid responsiveness and steroid dependence	Frequent	Less frequent	Less frequent
Long term visual recovery	Favourable in absence of subsequent relapses	May be poor	Favourable

*OCT* optical coherence tomography, *pRNFL* peripapillary retinal nerve fibre layer, *MOG* myelin oligodendrocyte glycoprotein, *MS* multiple sclerosis.

### Bilateral optic neuritis

Simultaneous, bilateral ON is a common onset presentation for MOG-ON, occurring in 31–84% of cases [7, 22, 42–45], but is rare in MS-ON [45, 46]. Bilateral ON is described in 13–82% of cases of AQP4-ON, noting variability in sample sizes across studies. Overall, the literature favours a higher frequency of bilateral ON reported in MOG-ON compared to AQP4-ON [44–46].

### Optic disc swelling

The presence of severe optic disc swelling and peripapillary haemorrhage in the setting of ON is highly suggestive of MOG-ON. Optic disc swelling visible on fundoscopy has been reported in 45–92% of cases of MOG-ON across various international cohorts [22, 43–45, 47–52], and is an important diagnostic clue when assessing patients with ON. This is far more common than the frequencies reported in AQP4-ON (7–52%) [44, 48, 50, 51], and MS-ON (11–14%) [49, 51]. Optic disc swelling is moderate to severe in many cases of MOG-ON and has been associated with peripapillary haemorrhage [43, 46]. MOG-ON with bilateral optic disc swelling may be mistaken for idiopathic intracranial hypertension, which affects a similar age group and is more common, though does not usually cause the rapid and substantial vision loss or pain on eye movement seen in MOG-ON [53]. MOGAD can also cause papilloedema secondary to raised intracranial pressure, in association with an aseptic meningoencephalitis presentation [54, 55].

### Eye pain

Eye pain may be common in MOG-ON, reported in 73–92% of cases internationally [42, 43, 48, 51, 56, 57]. In comparative studies, this proportion has been shown to be greater than in AQP4-ON (28–50%) and seronegative ON, including MS (10–46%) [48, 51, 58]. The majority of eye pain in MOG-ON is pain related to eye movement, with periocular pain being less common [56, 57]. Pain on eye movement may arise from traction on the common tendinous ring caused by retro-orbital pathology, whereas periocular pain is presumed to be governed by the trigeminal nerve. Both types of pain have been shown to be more common when the intra-orbital segment of the optic nerve is inflamed, as is often the case in MOG-ON, and less common when inflammation is restricted to the canalicular or intracranial segments [45, 59]. One study also showed that eye pain occurred more frequently in patients with MOG-ON who had optic nerve sheath enhancement on MRI than in those without, suggesting that spread of inflammation from the optic nerve to the surrounding meningeal nerve sheath containing nociceptive fibres from the trigeminal nerve may be responsible for pain [60]. Associated eye pain can be severe enough to result in a characteristic headache, as reported in adults, which can extend from the ocular region to the periorbital and fronto-temporal areas [61]. Pain usually precedes vision loss, by a median of three days in one study, but does not appear to be related to the severity of visual loss itself [57].

### Visual acuity at nadir

Visual acuity deficits at nadir in MOG-ON are generally moderate-to-severe, often logMAR 1.0 (Snellen equivalent 6/60) or worse [1, 43, 57]. Many studies comparing visual acuity and associated visual functional system scores at follow up in MOG-ON and AQP4-ON have shown worse outcomes in AQP4-ON [45, 51, 58], though rates of severe vision loss at nadir, defined as logMAR >1.0 seem to be similar in both conditions [48, 62]. Chen et al. [57] found that visual acuity tended to worsen for a median of four days from onset before reaching nadir in MOG-ON. It is important to note, however, that visual acuity can rarely be normal in MOGAD, in the case of optic perineuritis with sparing of the optic nerve itself [63, 64].

### Colour vision loss and relative afferent pupillary defect

Red desaturation and a relative afferent pupillary defect (RAPD) are common findings in ON due to any cause, and in limited studies have been identified in patients with MOG-ON [56]. However, it is important to note that in MOG-ON, where bilateral disease is common, a RAPD may be absent with bilateral, or previous contralateral, optic nerve involvement.

### Associations with other ophthalmic presentations

Aside from ON, various other ophthalmic presentations have also been reported in association with MOGAD. These include serum MOG IgG reported in association with uveitis [63, 65], peripheral ulcerative keratitis [63], acute macular neuroretinopathy [66], serous retinal detachment [67], venous stasis retinopathy [68], pre-retinal macular haemorrhage [69], orbital inflammatory syndrome [70] and orbital apex syndrome [71]. Nearly all cases were accompanied by intercurrent, typical MOG-ON, with retinal and orbital manifestations believed to be secondary to the spread of adjacent inflammation and oedema from the optic nerve and nerve head. The association of typical MOG-ON in association with these more atypical ophthalmic findings is important to note. It is not advisable to associate atypical ophthalmic presentations with the presence of low-titre or fixed cell based assay detection of MOG-IgG, as sensitivity and specificity in a clinical context with a low pretest probability is likely to dilute the clinical relevance of this biomarker. Prominent retinal changes are uncommon in MOG-ON and not reported in AQP4-ON but the association between uveitis and MS is well established [72]. An important distinction, is that uveitis in MOGAD has been reported as being anterior [63, 65], posterior [63], or intermediate [34, 63], whereas MS-associated uveitis is predominantly intermediate [73].

### Non-ophthalmic clinical associations

It is important to note that the onset of ON in MOGAD may also coincide with other typical MOGAD phenotypes, such as ADEM in children [74], and transverse myelitis in children and adults [20, 75]. Other phenotypes of MOGAD may also be recognised in association with ON, such as cerebral cortical encephalitis with seizures, aseptic meningitis and brainstem and cerebellar syndromes [76].

## Investigations

Ancillary investigations play an important role in the early diagnosis and management of ON. MRI is the imaging modality of choice to confirm the presence of optic neuritis and displays characteristic features in MOG-ON that can help distinguish it from other aetiologies. The results of other routine ophthalmological tests such as visual field testing or perimetry, optical coherence tomography (OCT) and visual evoked potentials (VEPs), as well as CSF analysis, are less specific in distinguishing aetiology but may still have a contributory role, particularly in confirming objective evidence of visual pathway involvement or the inflammatory nature of the disease (see Table 1).

### MRI

**Fig. 1:**
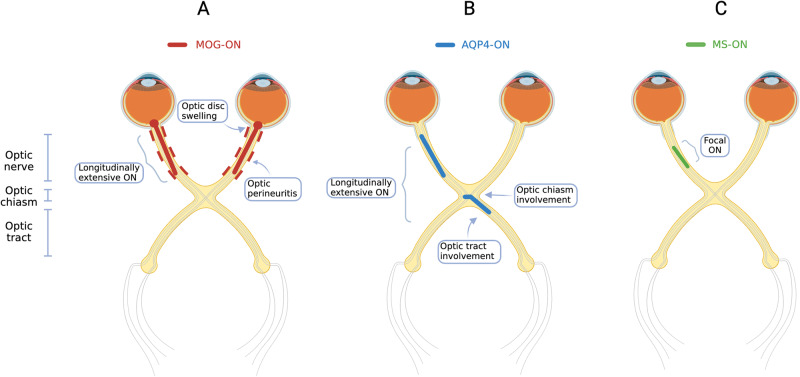
Localisation of visual pathway involvement in MOG-ON, AQP4-ON and MS-ON. **A** MOG-ON (red): Typical features include bilateral longitudinally extensive ON, with optic disc swelling, involvement of the retrobulbar segment of the optic nerve, and optic nerve sheath involvement or optic perineuritis. **B** AQP4-ON (blue): Longitudinally extensive ON, with optic chiasm +/− optic tract involvement. **C** MS-ON (green): focal ON. AQP4 aquaporin 4, MOG myelin oligodendrocyte glycoprotein, MS multiple sclerosis, ON optic neuritis.

**Fig. 2:**
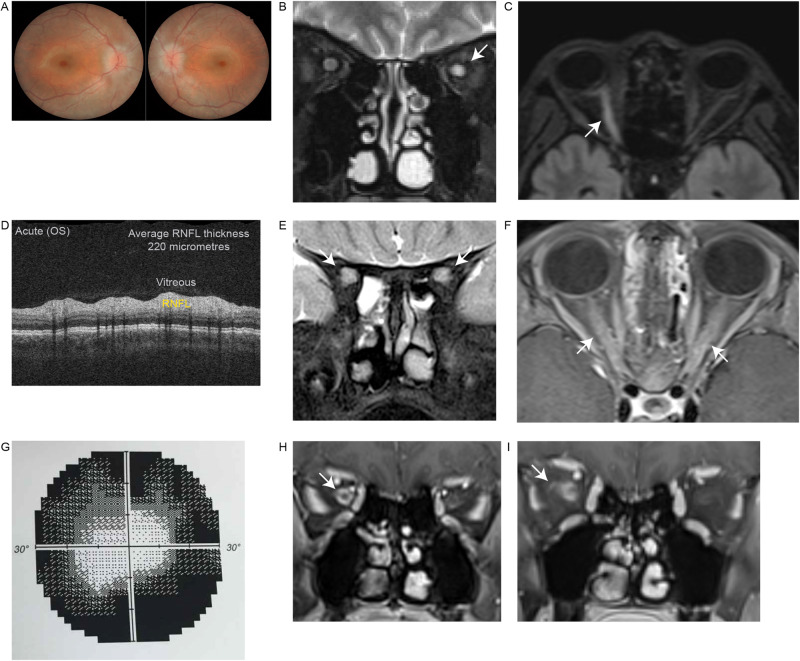
Clinical and radiological characterisation of MOG antibody-associated optic neuritis. **A** Bilateral optic nerve head swelling and oedema visualised on fundoscopy in a patient with a first presentation of MOGAD. **B** Unilateral left optic nerve T2 hyperintensity (arrow) (Cor T2). **C** Unilateral right sided longitudinally extensive optic neuritis (arrow) with associated T2 hyperintensity and swelling (Ax T2). **D** During acute left optic neuritis, swelling resulted in increased periparillary retinal nerve fibre layer (RNFL – yellow) thickness on optical coherence tomography. **E** Bilateral optic neuritis (arrows) with marked T2 hyperintensity (Cor T2 FS). **F** Bilateral longitudinally extensive optic neuritis (arrows) with gadolinium enhancement (Ax T1 FS Gad). **G** Visual field testing with dense peripheral constriction and relative central sparing in a pattern strongly suggestive of optic perineuritis. **H** Right optic perineuritis (arrow) with optic nerve sheath inflammation and enhancement (Cor T1 FS Gad). **I** Right optic perineuritis with extension of enhancement into intraorbital fat (arrow) (Cor T1 FS Gad). Figure 1A, G was reproduced from Ramanathan et al. [63] with permission from John Wiley and Sons. Ax axial, Cor coronal, FS fat suppressed, Gad gadolinium enhancement, MOGAD myelin oligodendrocyte glycoprotein antibody-associated disease, OS oculus sinister (left eye), RNFL retinal nerve fibre layer.

Optic nerve inflammation is best visualised on dedicated orbital MRI with T2 and T1 sequences with fat-suppression and gadolinium enhancement, and thin cuts of 1–3 mm these sequences should be specifically requested in the assessment of all patients with ON [77]. The presence of optic nerve enhancement is often present in ON and may distinguish it from non-inflammatory optic neuropathies [45, 78, 79]. Both gadolinium enhancement and T2 hyperintensity are present in many cases of MOG-ON, and T2 hyperintensity has been shown to be more prominent in MOG-ON and AQP4-ON than MS-ON [45]. Key radiological features of MOG-ON are described below and illustrated in Figs. 1 and 2.

#### Longitudinally extensive lesions

Longitudinally extensive optic neuritis is defined as ON involving more than 50% of the pre-chiasmal optic nerve length. This is characteristic of MOG-ON, occurring in 23–88% of patients, whereas it is rare in MS-ON [45, 77]. Whilst longitudinally extensive optic neuritis is also observed in AQP4-ON, in the range of 50–79%, longer segments are more frequently involved in MOG-ON [77].

#### Retrobulbar involvement

Multiple studies, including a recent meta-analysis, have established that MOG-ON most commonly affects the retrobulbar segment of the optic nerve [45, 79, 80]. In contrast, AQP4-ON tends to favour the more posterior canalicular and intracranial segments [80]. However, a review of comparative studies concluded that lesion location did not differentiate the two groups overall [77].

Reported rates of optic chiasm involvement vary widely between studies, likely owing to differences in sample size, and ranges from 5 to 17% in MOG-ON and 2 to 64% in AQP4-ON [45, 62, 81–83]. One study [45] detected a statistically significant difference between these rates in MOG-ON and AQP4-ON, whereas other studies did not find a significant difference [62, 81–83]. Importantly, chiasmal involvement, when it occurs in MOG-ON, is more likely to be part of longitudinally extensive disease extending up to the chiasm, in contrast to AQP4-ON where isolated chiasmal involvement can occur [81]. In MS-ON, chiasmal involvement is extremely rare.

Fewer studies have examined the rates of optic tract involvement, and whilst higher rates have been reported in AQP4-ON (4–13%) than MOG-ON (0-5%), this has not been shown to be statistically significant, likely due its rarity in general [62, 82, 83]. However, one study looking at bilateral optic tract involvement specifically, found this to be exclusive to AQP4-ON, where it was present in 45% of cases, compared to not being present in MOG-ON or MS-ON [45].

#### Optic disc swelling

While more commonly reported on fundoscopy as previously mentioned, or on optical coherence tomography (OCT), optic nerve head swelling can also be visualised on orbital MRI. Radiologically visible optic disc swelling is more common in MOG-ON than AQP4-ON and MS-ON [45, 80].

#### Optic perineuritis

Optic perineuritis is observed on MRI as circumferential, ‘tram-track’ enhancement of the optic nerve sheath, which may extend into the surrounding orbital fat [84]. This was first reported as a manifestation of MOGAD by Kim et al. in 2015 [85], and, since then, several other reports have emerged in the literature, both with [43, 63], and without [47, 64, 86], accompanying optic nerve involvement. MOGAD is now recognised as an important cause of optic perineuritis, alongside sarcoidosis, which remains the major differential diagnosis for this presentation [87]. Furthermore, very severe cases of MOG-related optic perineuritis leading to orbital apex syndrome, where there is involvement of multiple cranial nerves that traverse the orbital apex, leading to ptosis, proptosis and ophthalmoplegia, have been described [71, 88]. Interestingly, it has been hypothesised that focally elevated pressures in the subarachnoid space due to perineural inflammation may be responsible for the optic disc swelling in MOG-ON, linking these two features [86].

### Perimetry

A wide variety of non-specific visual field defects have been reported in MOG-ON including central and paracentral scotomas, temporal field cut and complete visual field loss [56, 89]. Perhaps the only distinctive pattern is that of peripheral vision loss with sparing of central vision, which can occur in MOG antibody-associated optic perineuritis, though this pattern is more commonly seen with alternate aetiologies such as raised intracranial pressure [63, 86]. At follow-up post-ON, the degree of visual field loss, measured as mean deviation from normal, has been reported to be better in MOG-ON than in AQP4-ON in one study [90] but not significantly different from MS-ON in another study [58].

### Optical coherence tomography

In the acute stages, MOG-ON leads to prominent thickening of the peripapillary retinal nerve fibre layer (pRNFL) on OCT, reflecting oedema. In fact, the amount of pRNFL thickening in acute MOG-ON has been shown to be significantly greater than in MS-ON (median pRNFL thickness 164 μm vs. 103 μm respectively), consistent with the more frequent observation of fundoscopic optic disc swelling and may be because of its unique predilection for the intra-orbital and longer segments of the optic nerve [91]. OCT may therefore have a place as an early diagnostic tool to differentiate MOG-ON from MS-ON. Chen et al. found that using a pRNFL thickness cut-off of 118 μm afforded a sensitivity and specificity for MOG-ON of 74% and 82% respectively, compared with MS-ON [91]. Similar comparisons have not yet been made with acute AQP4-ON.

Conversely, in the months following acute ON, thinning of the pRNFL as well as of the macular ganglion cell inner plexiform layer (GCIPL) occurs with significantly lower pRNFL and GCIPL thickness found in MOG-ON than in both healthy controls and in MS-ON according to a recent meta-analysis [92, 93]. However, no significant difference was observed in these parameters when compared to AQP4-ON [92].

Relapses may be the main driver of pRNFL atrophy in MOG-ON, with some studies demonstrating a positive correlation between the number of ON episodes and reduction in pRNFL thickness in MOG-ON but not in MS-ON or AQP4-ON [93, 94]. Meanwhile, Akaishi et al. showed that pRNFL thickness was not correlated with months post-ON in MOG-ON, suggesting that retinal thinning in MOG-ON may be primarily driven by acute relapses rather than chronic or progressive atrophy [95].

Furthermore, post-ON pRNFL thinning has also been shown to be linked to functional visual measures in MOG-ON such as worse mean visual field defects and visual acuity [58, 90]. Interestingly, however, functional outcomes such as these seem to be better in MOG-ON than in AQP4-ON despite similar structural damage as described earlier. This mismatch between structure and function may be related to differences in the histological composition of the various retinal layers in these pathologies, which is not captured by OCT, for instance due to differences in the glial population [92]. A floor effect may also be operative, whereby a few, severe attacks of ON are sufficient to lead to marked reductions in pRNFL and GCIPL thickness so that subsequent attacks do not cause appreciable additive atrophy but continue to worsen function [96]. Alternatively, there may be a threshold effect, with significant functional retinal reserve that is maintained despite accumulating structural damage, until a specific threshold. Deschamps et al. [97] found a threshold pRNFL thickness of 50 µm, below which mean deviation of the visual field was significantly worse. Indeed, clinical experience in MOGAD-ON demonstrates that patients may maintain favourable visual acuity after recovery despite pRNFL thinning following early ON episodes.

In addition to conventional OCT, OCT angiography has also emerged as a tool in MOG-ON, to evaluate the retinal microvasculature. Using this technique, reduced peripapillary and parafoveal vessel densities have been observed in MOG-ON compared to healthy controls [98, 99], though comparisons with AQP4-ON have had mixed results, with one study reporting similar vessel densities [98] and others reporting reduced densities in MOG-ON [99]. Furthermore, the reduction in vessel densities has been correlated with other parameters such as the number of ON episodes [100], pRNFL thickness and visual acuity [99]. The mechanism behind retinal vascular rarefaction in MOG-ON is unclear but may be related to reduced metabolic demand following retinal degeneration [99].

### Visual evoked potentials

VEPs in the majority of MOG-ON cases can demonstrate both delayed P100 latencies and reduced amplitudes, the former being more common [42, 94]. In severe cases, the evoked potential can be absent. Comparisons to multifocal VEPs in MS-ON and AQP4-ON in one study showed significantly longer latencies in MS-ON than MOG-ON but no significant difference in latencies between AQP4-ON and MOG-ON [93]. There was a trend towards lower amplitudes in AQP4-ON than MOG-ON but this did not reach statistical significance [93]. Overall, the main utility of VEP testing in MOG-ON may be in the confirmation of subtle optic nerve injury in cases where clinical examination findings may be uncertain, and in discriminating organic visual loss from functional visual loss, rather than in discriminating MOG-ON from other demyelinating aetiologies.

### CSF analysis

CSF findings in MOG-ON may be contributory but are neither specific nor sensitive for diagnosis. Pleocytosis, which predominantly comprises mononuclear cells, and can be seen in acute attacks, has been reported in 44–72% of patients with MOGAD [42, 43, 101]. CSF pleocytosis appears to be more common in patients with myelitis rather than ON, and very high white cell counts of greater than 50 cells/µL are nearly exclusive to myelitis presentations [101]. Similarly, elevated CSF protein, reflecting dysfunction of the blood-brain barrier, is seen in 32–42% of cases of MOGAD, including some presenting with ON, but is also more common in those with myelitis [43, 101]. The presence of CSF-restricted oligoclonal bands, classically a hallmark of MS, is less common in MOGAD, with reports ranging from 0 to 20% [42, 43, 101]. One potentially differentiating feature is the so-called ‘MRZ reaction’ defined by the presence of intrathecal antibodies to at least two out of three of the measles, rubella and varicella zoster viruses, which has been shown to be common (over 60%) in MS but so far absent in MOGAD [42, 101], although this is not a test that is widely performed. CSF metabolomics and chemokine and cytokine profiles, though non-specific in MOG-ON, may have future use in differentiating MOG-ON from non-inflammatory causes [25].

### MOG IgG testing

The detection of serum MOG IgG using a live cell-based assay is highly specific and sensitive for a diagnosis consistent with MOGAD [1]. In contrast, fixed cell-based assays have lower sensitivity and specificity [102–104]. Serum is the recommended biospecimen for testing for MOG IgG. While CSF may be positive for MOG IgG in 40-60% of patients with MOGAD, in many patients the diagnosis would be missed if CSF was tested in isolation [105, 106].

Given that up to 50% of children presenting with demyelination under the age of 10 years will have MOGAD as a final diagnosis, all children in this age group with a first presentation consistent with demyelination, including optic neuritis, should be tested for MOG IgG [1]. In contrast, MOG IgG serological testing should only be performed in adults with demyelination in the context of a suspected clinical or radiological phenotype for MOGAD. Testing large numbers of unselected patients with likely MS-ON in the context of typical MS brain lesions who have a low pre-test probability for MOGAD, will stretch the specificity for even the most specific and sensitive diagnostic biomarker [107].

Finally, interpretation of CSF-restricted MOG IgG (for example in a seronegative patient with ON) requires clinical acumen. While this may be contributory to the diagnosis in seronegative cases where there is a clinicoradiological profile highly associated with MOGAD, in atypical cases this could represent a false positive result [1, 105, 106, 108, 109].

## Diagnosis

Diagnostic criteria for MOGAD were recently proposed [1]. ON is considered to be one of the core clinical demyelinating events necessary for the diagnosis of MOGAD, though no distinction is made between definite and possible ON as defined in the recently proposed diagnostic criteria for ON by Petzold et al. [110] In a patient with ON, if clear positive serum MOG IgG antibodies are then detected on a cell-based assay and a better alternate diagnosis is excluded, the diagnosis of MOG-ON can be made. Where there is only a low positive serum MOG IgG, a positive result without a reported titre, or CSF restricted MOG IgG antibodies only, then at least one supportive clinical or radiological feature (bilateral simultaneous involvement, >50% length involvement, perineural optic sheath enhancement or optic disc oedema) and negative serum aquaporin-4 antibodies are additionally required to reduce the likelihood of misdiagnosis, and increase the specificity of conferring a diagnosis of MOGAD.

## Differential diagnoses

In addition to AQP4-ON and MS-ON, a range of other conditions can also present with ON and must be considered in the differential diagnosis of MOGAD [111]. These are outlined below and summarised in Table 2.

**Table 2:** Differential diagnoses of MOG-ON.

Differential diagnosis	ON characteristics	Other neurological involvement
**Immune mediated**		
NMOSD	Longitudinally extensive, may be bilateral, posterior segment, can have chiasmal +/− optic tract involvement	Longitudinally extensive transverse myelitis, area postrema syndrome, hypothalamic lesions
Multiple sclerosis	Focal involvement	Transverse myelitis; typical supratentorial, periventricular, brainstem and cerebellar involvement
GFAP antibody	Bilateral optic neuritis, optic disc swelling	Meningoencephalitis, longitudinally extensive transverse myelitis
SOX2 antibody	A chronic relapsing inflammatory optic neuropathy	Not specified
GlyR antibody	ADEM-ON, a chronic relapsing inflammatory optic neuropathy	Progressive encephalitis with rigidity and myoclonus
**Paraneoplastic**		
CRMP5 antibody	Bilateral optic neuritis with retinitis and vitritis	Limbic encephalitis, cerebellar ataxia, myelopathy, peripheral neuropathy
Recoverin antibody	Bilateral optic neuritis, optic disc swelling	Variable
**Systemic**		
Sarcoidosis	Optic perineuritis, bilateral optic neuritis, optic disc swelling	Cranial neuropathies, leptomeningeal involvement, myelopathy, parenchymal disease, muscle disease, peripheral neuropathy
Systemic lupus erythematosus	Variable	co-existing NMOSD; longitudinally extensive transverse myelitis
Others: Behcet, ANCA vasculitis	Variable	Variable
**Infection** e.g. *T. pallidum, B. henselae, B. burgdorferi, herpes simplex* viruses*, varicella zoster virus, cytomegalovirus*	Variable	Variable
**Genetic**		
Leber hereditary optic neuropathy	Bilateral, painless, subacute	‘Leber hereditary optic neuropathy-plus’ – movement disorders, MS-like illness
**Vascular**		
Non-arteritic ischaemic optic neuropathy	Optic disc swelling	Cerebrovascular disease

*NMOSD* neuromyelitis optica spectrum disorder, *GFAP* glial fibrillary acidic protein, *GlyR* glycine receptor, *ADEM-ON* acute, disseminated optic neuritis-optic neuritis, *CRMP5* collapsin response mediator protein 5.

### Other autoantibody-associated ON

#### Glial Fibrillary Acidic Protein (GFAP) antibody-associated ON

Autoimmune GFAP astrocytopathy is another inflammatory disease of the CNS and typically presents as a meningoencephalitis associated with GFAP-IgG antibodies in the CSF and a characteristic radial, perivascular enhancement pattern on brain MRI [112]. Rarely, in about 6% of all cases, it can present with ON. Like MOG-ON, these patients may also present with bilateral optic disc oedema with normal CSF opening pressures, though the mechanism in this scenario is thought to be related to primary venulitis with prominent vascular leakage [113].

#### Collapsin Response Mediator Protein 5 (CRMP5) antibody-associated ON

CRMP5-IgG antibodies are associated with a wide range of phenotypes affecting the peripheral and central nervous system including peripheral neuropathy, myelopathy, cerebellar ataxia and optic neuritis, and are typically paraneoplastic in origin, with small cell lung cancer being the most frequently identified tumour [114]. ON is a presentation in some patients with this condition, for whom bilateral optic disc oedema is characteristic, but specific to this condition, is typically accompanied by retinitis and vitreous inflammatory cells [115].

#### Other autoantibodies

Various other autoantibodies have also been linked with ON, though evidence for their association is less robust. For example, antibodies to recoverin, the photoreceptor protein, which are typically found in paraneoplastic retinopathy have been reported in a case of ON [116]. Additionally, antibodies to SOX2, a transcription factor expressed by glial cells of the optic nerve, have been isolated in a subset of patients with relapsing inflammatory optic neuropathy [117]. Furthermore, anti-glycine receptor antibodies, typically associated with the syndrome of progressive encephalomyelitis with rigidity and myoclonus, have been linked to cases of demyelinating optic neuritis, although these results have not been clearly reproduced [118].

### ON associated with systemic conditions

#### Sarcoidosis

Sarcoidosis is a multisystem autoinflammatory disorder characterised by non-caseous granuloma formation. Ophthalmological manifestations occur in 10–50% of patients, with anterior uveitis being most common [119]. Neurological involvement, which can be peripheral or central and can include ON, is less common and occurs in 5–10% of patients, but is noteworthy because it shares similarities with MOG-ON [120]. For instance, sequential, bilateral involvement may be present in sarcoidosis-related ON, occurring in 28% of cases in one major published case series, although synchronous disease is rare and occurs in only 9% [119]. In the same series, 37% also had visible optic disc swelling and 4% had optic perineuritis. The proportion of cases of optic perineuritis due to sarcoidosis is higher still, at 20% in the largest published case series, and trails only MOGAD as the major secondary cause of optic perineuritis, though idiopathic cases are also well recognised [87]. The finding of non-caseous granulomas on biopsy definitively distinguishes sarcoidosis from MOG-ON, as well as multisystem involvement of sarcoidosis.

#### Other systemic autoimmune conditions

Many other systemic autoimmune conditions have also been reported to feature ON, including Sjogren’s syndrome, Behcet’s disease, ANCA vasculitis, and systemic lupus erythematosus, the latter often occurring in association with AQP4 IgG antibodies [111, 121].

#### Infection

Infectious causes of ON include bacteria such as *T. pallidum, B. henselae* and *B. burgdorferi* and viruses such as *herpes simplex* viruses*, varicella zoster virus* and *cytomegalovirus* [122]. Importantly, as mentioned earlier, MOG-ON has been reported as occurring alongside, or being triggered by, a number of these infectious aetiologies [34, 35].

### Leber hereditary optic neuropathy

Leber hereditary optic neuropathy is a mitochondrial disorder preferentially affecting young males that is characterised by degeneration of the retinal ganglion cell layer, leading to painless, subacute, bilateral central vision loss. Symptom onset is usually in the second or third decade of life, following which occurs progression to visual acuity of 6/60 or worse, over four months on average in one series; with sequential, contralateral eye involvement by two months on average [123]. Thus, Leber hereditary optic neuropathy at first presentation may be considered as a differential for MOG-ON, but the presence of pain and the more rapid visual loss in MOG-ON forms a key distinction. Interestingly, there is one reported case of acute MOG-ON occurring in a known Leber hereditary optic neuropathy carrier [124].

### Chronic relapsing inflammatory optic neuropathy

In 2003, Kidd et al. first described a syndrome of unilateral or bilateral, painful, subacute vision loss with initial steroid responsiveness and tendency to relapse on cessation of steroids, termed chronic relapsing inflammatory optic neuropathy (CRION) [125]. Following the discovery of AQP4 IgG and MOG IgG, serum antibody testing of original CRION cohorts demonstrated that subgroups of these patients were in fact seropositive for one of these two antibodies, with up to 22% being AQP4 IgG positive and up to 25% MOG IgG positive [126–128]. This is not surprising given that CRION is by definition a syndromic diagnosis, and one of exclusion, likely comprising a heterogenous group of underlying aetiologies that includes MOG-ON and AQP4-ON.

## Disease course, outcomes and relapse prediction of MOGAD

While early reports with median follow-up times of less than two years suggested that MOGAD might be a predominantly monophasic illness [21, 129], a few studies examining patients over longer durations of more than five years have demonstrated relapse rates of up to 70% [42, 130].

It should be considered that patients with disease onset prior to the widespread availability of the MOG IgG antibody test are likely to only be diagnosed with MOGAD if they re-present with relapsing disease, meaning that relapsing patients may be over-represented in retrospective cohort studies [6]. There have only been a few incident studies to address this bias and the largest and longest of these reported a 4-year relapse risk of 32% but included more children, who may have a lower risk of relapse than adults [75, 131]. However, when considering only cases of ON at onset, Cobo-Calvo et al. found no significant difference in relapse rates between children and adults although group means were not reported [75].

ON is the most common relapsing syndrome in both adults and children with MOGAD, occurring in 28-91% of relapsing cases, and may be recurrent unilateral, contralateral or simultaneous bilateral ON [6, 7, 42, 131, 132]. Furthermore, patients with ON at onset may be more likely to develop relapsing disease than those with other clinical presentations, and relapsing ON may be associated with more attacks than other relapsing phenotypes [6, 130]. Patients with ON at onset can also develop other, non-ON relapsing syndromes such as transverse myelitis [131], aseptic meningitis [54] and cerebral cortical encephalitis with seizures [133].

In children, a distinct relapsing phenotype of monophasic ADEM or recurrent/multiphasic ADEM (MDEM) followed by ON after at least three months, termed ADEM-ON, is well recognised [134]. Though ADEM-ON may be rare overall when considering all acquired demyelinating syndromes [135], it is seen in up to 40% of children with relapsing MOGAD with an initial presentation of ADEM [7, 74]. A median of three relapses per patient over a median follow-up time of 5.3 years, of which 94% of relapses are ON, has been described in one ADEM-ON cohort [134]. Importantly, unlike the high level of functional recovery previously reported in children [75], 71% of ADEM-ON patients in this cohort had residual deficits, most commonly visual impairment.

MOGAD patients at disease onset may be prone to ‘very early relapses’ (<3 months after onset) and ‘delayed early relapses’ (3–12 months after onset). Chen et al. demonstrated that very early relapses in adults, and delayed early relapses in children and adults, were associated with an increased risk of long-term relapsing disease [136]. This provides a compelling reason to consider whether therapeutic choices at disease onset have the potential to modify natural history of MOGAD.

Overall, despite frequent relapses, patients with MOGAD are reported to have comparatively favourable outcomes, with expanded disability status scale (EDSS) scores of 1–2 reported at last follow-up [7, 130], compared to AQP4-IgG positive NMOSD where follow-up EDSS can often be >3 [21, 129, 137]. However, it is important to note that the EDSS was developed for use in classical MS and is strongly weighted towards locomotor deficits, making visual disability likely to be underestimated. In one study, severe visual impairment, defined as visual acuity worse than 50% of normal in one or both eyes, was present at last follow-up in 36% of patients with MOGAD [7, 42] whereas other studies have reported lower rates of 16–24% [6, 7, 138]. Unlike in MS, thus far, only relapse-related stepwise progression of disability has been observed in MOGAD, without clear evidence to support relapse-independent progression [139].

A means to predict relapse is of significant interest. A number of studies have observed the conversion of MOG IgG to seronegativity over time in monophasic patients, whereas relapsing patients tend to remain persistently MOG IgG seropositive but with lower titres during periods of remission than during active disease [130, 140]. However, many patients who display persistently positive MOG IgG can still remain monophasic [3, 6] and, conversely, albeit rarely, patients can still relapse after seroconversion, possibly related to assay sensitivity or patient treatment timing relative to serological testing [137]. Prospective studies are needed to elucidate the exact role of MOG IgG titres in predicting relapsing disease. Recent publications identified that adults with MOG IgG recognising a non-proline 42 epitope were at a higher risk of relapse, suggesting that epitope profiling at disease onset may also assist with prediction of a relapsing course [103, 141].

## Treatment

### Acute therapy

The use of high dose intravenous methylprednisolone for 3–5 days is common practice internationally for the treatment of acute attacks of ON [142]. This is in line with the results of the landmark Optic Neuritis Treatment Trial (ONTT) evaluating 448 adults with ON and supporting intravenous methylprednisolone, though only three of the patients in this study of a subgroup of 177 in whom archival sera was available for testing, were subsequently found to be seropositive for MOG IgG [143, 144]. In a large, retrospective study of MOGAD patients by Jarius et al. [42], intravenous methylprednisolone treatment was associated with complete recovery in half and partial recovery in 44% of patients.

MOGAD appears to be highly steroid sensitive, with rapid recovery following initial administration of intravenous and/or oral corticosteroids [7, 22], but also to some extent steroid dependent. In one study, 70% of episodes treated with oral prednisone relapsed, particularly at doses of <10 mg a day in adults (or a weight based equivalent in children) or within two months of cessation, cautioning against rapid tapers [7]. A number of further studies have confirmed the modulating effect of oral corticosteroids on inducing disease remission, with suggested treatment durations ranging from five weeks to six months, and significant variability depending on clinician experience and preference, highlighting that this is a matter of notable equipoise [6, 42, 142, 145]. Identifying the minimum dose and duration of corticosteroid treatment at disease onset which will delay time to first relapse, while limiting steroid related adverse effects, will be a significant step forward in the acute management of this condition.

In terms of treatment timing, after adjusting for initial visual acuity, Chen et al. [57] found no difference in outcomes between MOG-ON patients treated with intravenous methylprednisolone within 3 days of onset and those treated later, however, better outcomes were reported in a smaller cohort with treatment initiation prior to 7 days [146]. Rode et al. found that treatment initiation after 10 days was significantly associated with failure to recover visual acuity at three months and reduced pRNFL thickness (i.e. structure and function) [147].

There is limited data on the efficacy of second-line agents for acute attacks, such as intravenous immunoglobulin and plasmapheresis, when the initial response to steroids is poor, but this scenario is less common [42, 57]. In a survey of international experts on MOGAD, the most popular second-line agent was plasmapheresis, with 81% of respondents in favour and others preferring to retrial high dose corticosteroid treatment [142]. Certainly, intravenous immunoglobulin and plasmapheresis are well established first line therapeutic agents in neuroglial cell surface antibody syndromes including NMOSD and autoimmune encephalitis, and likely to be of benefit in MOGAD.

### Maintenance therapy

There are no published randomised, controlled clinical trials of treatment to prevent relapses in MOGAD, although such studies are currently recruiting for novel immunotherapeutic agents.

Importantly, short ‘pulses’ of high dose corticosteroids alone appear to be insufficient for lasting disease activity control following acute MOG-ON. In one cohort, 70% of MOGAD episodes treated with oral prednisolone relapsed, particularly at doses under 10 mg or within two months of cessation [7]. Another study found similar results and also showed that the risk of relapse was significantly greater in patients with corticosteroid treatment durations of less than three months compared to those that received more [6]. However, there are heightened concerns about prolonged steroid tapers, especially in children, because of the neuropsychiatric, metabolic and infection risks associated with long-term steroid exposure [136, 148].

The role of steroid-sparing maintenance therapy after the first acute MOG-ON attack is unclear. In one study, maintenance steroid therapy was superior in efficacy to non-steroidal maintenance immunotherapy, including intravenous immunoglobulin, rituximab and mycophenolate, with respect to relapse prevention [7], however concerns related to the long-term side effects of steroid based regimes make this less viable as a long-term maintenance treatment option. A large, international retrospective cohort study found that maintenance intravenous immunoglobulin treatment was associated with a significant reduction in annualised relapse rates of MOGAD, and displayed a dose-response relationship with patients who received at least 1 g/kg of intravenous immunoglobulin every 4 weeks experiencing significantly less relapses than those who received lower or less frequent doses [149]. The role of rituximab in relapse prevention for MOGAD remains less clear. Evaluation of rituximab in MOGAD showed that it reduced relapse rates, though some patients still experienced relapses despite apparent B-cell depletion [150]. In a prospective study, a significant proportion of MOGAD relapses on rituximab treatment occurred despite effective B-cell depletion compared to AQP4-IgG positive NMOSD relapses, implicating less biological efficacy in MOGAD [151]. A meta-analysis of nineteen studies found a significantly greater reduction in annualised relapse rates post-rituximab in AQP4-IgG positive NMOSD compared to MOGAD [152].

Other commonly used immunomodulatory agents such as methotrexate, azathioprine and cyclophosphamide have also been retrospectively studied in MOGAD, with benefits when compared to a ‘no treatment’ group, but none have been shown to be completely effective in preventing relapses [42]. Traditional disease-modifying therapies used in MS do not appear to be effective in MOGAD, and may exacerbate disease, as can be seen in NMOSD [42, 131, 153]. There is no clear hierarchy amongst these different steroid-sparing agents. However, switching maintenance immunotherapy following treatment failure, irrespective of the initial agent, has been shown to reduce relapse rates in MOGAD, supporting a dynamic and multifaceted treatment approach which may need to be individualised [7].

Relapse prevention is a key goal in the management of MOGAD as relapse-associated disability, particularly in relation to visual (following ON) and sphincter (following TM) dysfunction [6, 42]. While relapse rates are reported as 30–60% in MOGAD, this may increase to the order of up to 70% when patients are followed up for over five years [42]. This brings to discussion whether there is a role for introducing corticosteroid-sparing maintenance immunosuppression at ON onset. However, while the treatment of MS and NMOSD highlights the need for ongoing immunotherapy from diagnosis, in MOGAD there remains a proportion of patients who have a monophasic illness who may be unnecessarily exposed to long-term immunosuppression if this approach was uniformly adopted at disease onset.

Whilst the therapeutic landscape remains under investigation, novel agents including monoclonal antibodies targeting the neonatal Fc receptor (rozanolixizumab; NCT05063162) and IL-6 receptor (satralizumab; NCT05271409) are currently undergoing phase III double blind placebo-controlled randomised controlled clinical trials.

## Conclusion

MOG-ON is an increasingly recognised condition affecting both children and adults that deserves specific attention because of its distinction from ON associated with MS and NMOSD, and the therapeutic and prognostic implications of an early and accurate diagnosis. Characteristic clinical and radiological features can expedite diagnosis and facilitate initiation of appropriate therapeutic pathways. Confirmation of the diagnosis ideally relies on the detection of specific serum MOG IgG antibodies on a live cell-based assay, in association with a typical clinical phenotype. Although a relapsing disease course is common, and outcomes with treatment can be favourable, residual disability may be present. Future research priorities include better elucidating the underlying pathophysiological mechanisms in this condition, identifying prognostic biomarkers, and establishing optimal and well-tolerated long-term therapeutic strategies.

## Data Availability

As this is an invited review, there is no original data. All sources have been referenced.
